# Relationship between glycemic Lability Index, infections and outcome in critically ill patients

**DOI:** 10.1186/cc10773

**Published:** 2012-03-20

**Authors:** A Donati, L Botticelli, R Castagnani, V Gabbanelli, E Damiani, R Domizi, P Pelaia

**Affiliations:** 1 Università Politecnica delle Marche, Ancona, Italy

## Introduction

Hyperglycemia and glucose variability are important factors associated with morbidity and mortality in critically ill patients [1,2]. Our objective was to determine the association between the glucose Lability Index (LI), infections and outcome in critical illness.

## Methods

We performed a retrospective study in 2,943 adult patients admitted to our ICU from 2004 until 2010. Glucose variability was calculated for all subjects as the LI [3] during the hospital stay on capillary, arterial and venous blood. The ROC curve was performed to verify discrimination of the LI towards mortality and ICU infections.

## Results

**Figure 1:**
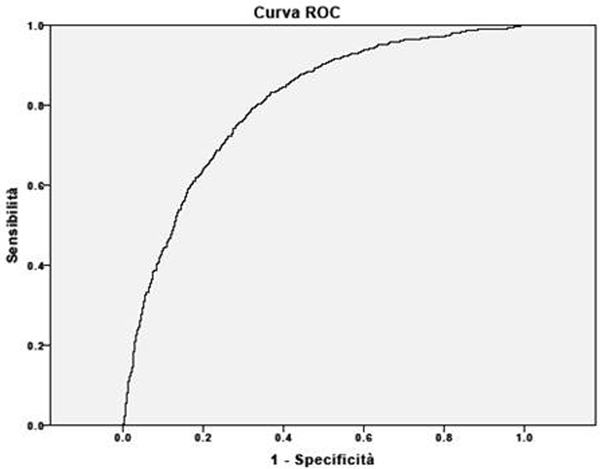


**Figure 2:**
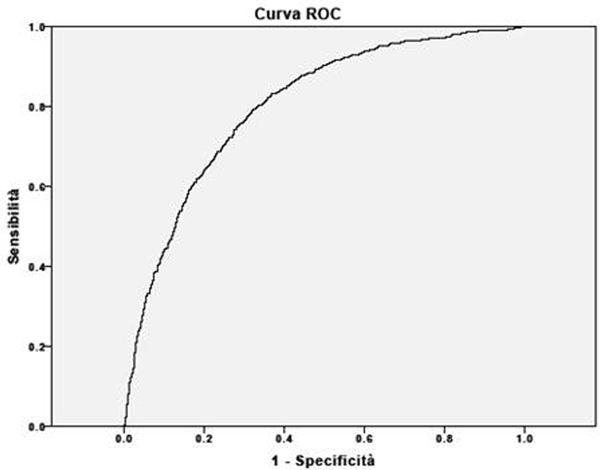


There were 709 infections and 447 deaths. There was a significant interaction between the LI and infections in patients. The LI had a great ability to predict hospital mortality (area under the curve = 0.62, 95% CI = 0.59 to 0.65, *P *< 0.5; Figure 1) but moreover infections (area under the curve = 0.80, 95% CI = 0.78 to 0.82, *P *< 0.5; Figure 2).

## Conclusion

Glucose variability has ability to predict outcome but moreover infections in patients in the ICU, because it is a predictor of clinical outcomes in patients with hyperglycemia and diabetes. Strategies to reduce glucose variability should be studied to improve the outcomes in ICU patients.
